# Subjective Visual Vertical in PD Patients with Lateral Trunk Flexion

**DOI:** 10.1155/2016/7489105

**Published:** 2016-03-17

**Authors:** F. Gandor, D. Basta, D. Gruber, W. Poewe, G. Ebersbach

**Affiliations:** ^1^Movement Disorders Clinic, Strasse nach Fichtenwalde 16, 14547 Beelitz-Heilstätten, Germany; ^2^Department of ENT, Trauma Hospital Berlin, Warener Strasse 7, 12683 Berlin, Germany; ^3^Department of Neurology, Medical University of Innsbruck, Anichstrasse 25, 6020 Innsbruck, Austria

## Abstract

Lateral trunk flexion (LTF) is a common phenomenon in patients with Parkinson's disease (PD) and has recently been associated with peripheral vestibular dysfunction. Since deviation of the subjective visual vertical (SVV) is a well-recognized feature of disorders involving vestibular processing, we analyzed SVV angles in 30 PD patients with and without LTF to assess the possible role of vestibular dysfunction in the pathogenesis of LTF in PD. Quantification of SVV was obtained using a simple bedside test. PD patients with LTF had significantly greater SVV angles as compared to PD patients without LTF (median: 4.3° [range: 0.1–17.7], *n* = 21, versus 0.8° [0.1–1.9], *n* = 9; *p* < 0.001). 14 of 21 patients with LTF showed pathological SVV, while all 9 patients without LTF had normal SVV. Abnormal SVV was more frequent when LTF was reversible in the supine position compared to fixed LTF. In a subgroup of PD patients with LTF, pathological SVV suggests vestibular dysbalance, which might be involved in the pathophysiological mechanisms underlying LTF.

## 1. Introduction

Lateral trunk flexion (LTF) in PD is usually evident when subjects are standing or sitting and may be reversible in the supine position. The term “Pisa Syndrome” has been defined as LTF > 10° in the standing position, which typically subsides in the supine position (“mobile LTF”) [1]. However, Doherty et al. proposed extending the definition of Pisa Syndrome to “fixed LTF” where spinal curvature persists when lying down [2]. Recently, Vitale and colleagues have provided evidence for peripheral vestibular dysbalance in PD patients with LTF applying vestibular tests including caloric testing, head-shaking test, vibration test, and fast positioning maneuvers [3]. Since vestibular dysbalance in the roll plane is associated with deviation of the subjective visual vertical (SVV) [4], we applied a simple bedside test to measure SVV in PD patients with or without LTF in an attempt to obtain further evidence for vestibular dysfunction in LTF of PD.

## 2. Methods

### 2.1. Patients

This study was approved by the regional Ethics Committee and conducted in accordance with the 1964 Declaration of Helsinki and its later amendments.

Between April 2012 and June 2013, 24 consecutive PD patients with LTF of at least 10° were screened. Inclusion criteria were comprised of Parkinson's disease according to the Queen Square Brain Bank criteria and LTF of at least 10° or no LTF for the control group. Exclusion criteria included history of vestibular disease, cerebrovascular events, or spinal surgery. LTF angles were measured using the iPhone® application Angle Meter [5]. Three PD patients with LTF had a history of ischemic stroke and were excluded. Hence, 21 PD patients with LTF and 9 age-matched PD patients without LTF were included (Table 1). The primary outcome of interest was to identify a difference in SVV angles when comparing PD patients with LTF to PD patients without LTF.

### 2.2. Subjective Visual Vertical

**Figure 1:**
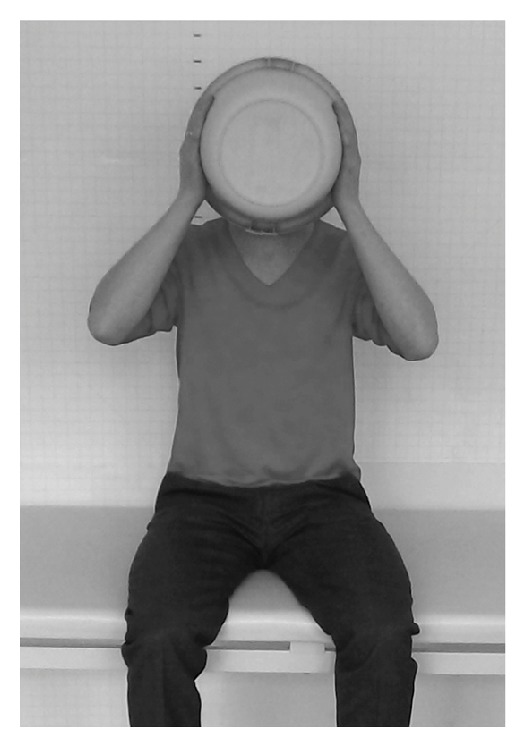
Testing the subjective visual vertical with the adapted bucket method.

SVV was assessed as described elsewhere [6] with the following changes: the iPhone application Visual Vertical [7] was applied. An iPhone was fixed to the bottom of a bucket. The application shows a red line on the screen for 15 seconds. For assessment of SVV, the bucket was randomly rotated left or right and handed over to the patient, who was then asked to align the red line on the screen into the supposedly vertical position within 15 seconds. The SVV angle is detected by the angle meter of the iPhone and shown on the screen after the test period. This test was performed three times per patient in a seated position (Figure 1), and mean deviation of absolute SVV values was calculated. Patients were assessed under regular dopaminergic treatment and, in case of response fluctuations, during the on-state. The cut-off value for SVV deviation from normal was set at 0 ± 2.5°, according to published values in healthy controls [8].

### 2.3. Data Analysis

SVV angles in PD patients with and without LTF were compared applying Mann-Whitney *U* test. Demographic and clinical features were compared on an exploratory basis. Distribution was calculated applying Shapiro-Wilk test, and variance was calculated using *F*-test. Categorical data were compared with Chi square test and Fisher's exact test, quantitative data were compared with *t*-test for heterogeneous variances for normally distributed values (Welch's *t*-test) and Mann-Whitney *U* test for not normally distributed values. Alpha-level was set at 0.05.

## 3. Results

**Figure 2:**
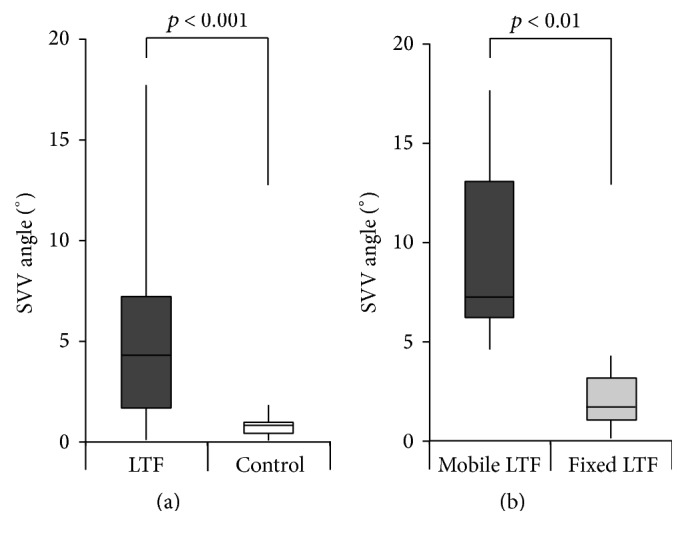
Comparison of SVV angles. (a) Comparison of SVV angles of PD patients with LTF (dark grey) and without LTF (control, white) (4.3° [range: 0.1–17.7], *n* = 21, versus 0.8° [0.1–1.9], *n* = 9). (b) Comparison of SVV angles of all PD patients with mobile LTF (dark grey) and all PD patients with fixed LTF (light grey) (7.3° [4.6–17.7], *n* = 10, versus 1.7° [0.1–4.3], *n* = 11).

PD patients with LTF had significantly greater SVV angles as compared to PD patients without LTF (median: 4.3° [range: 0.1–17.7], *n* = 21, versus 0.8° [0.1–1.9], *n* = 9; *p* < 0.001, Figure 2(a)). 14 of 21 PD patients (67%) with LTF had pathological SVV angles outside the normal range of ±2.5°, while all 9 PD patients without LTF showed normal SVV angles (Table 1). In 10 PD patients with LTF, trunk flexion subsided when lying down (mobile LTF), while the remaining 11 patients had persistent LTF in the supine position (fixed LTF).

At the subgroup level of cases with LTF, all PD patients with mobile LTF had pathological SVV angles, whereas this was found in only 4 of 11 PD patients (36%) with fixed LTF (Table 1). PD patients with mobile LTF had significantly greater SVV angles as compared to PD patients with fixed LTF (7.3° [4.6–17.7], *n* = 10, versus 1.7° [0.1–4.3], *n* = 11; *p* < 0.01, Figure 2(b)). Furthermore, when only comparing patients with pathological SVV deviation, SVV angles were again significantly greater in PD patients with mobile LTF in comparison to those with fixed LTF (7.3° [4.6–17.7], *n* = 10, versus 3.6° [2.8–4.3], *n* = 4; *p* < 0.05).

10 of 14 PD patients (71%) with pathological SVV but none with normal SVV reported LTF to have occurred after PD symptom onset. All PD patients with mobile LTF reported lateral flexion occurring after PD symptom onset, and in all PD patients with fixed LTF, lateral flexion was reported to have occurred before PD symptom onset (Table 1).

Absolute angles of lateral trunk deviation did not significantly differ between PD patients with SVV angles inside or outside the normal range (10° [10–21] versus 14° [10–48]; *p* = 0.56, Table 1). The majority of PD patients with LTF had flexion to the side of PD symptom onset (13 of 21, 62%), and SVV was deviated towards the side of LTF in 12 of 14 LTF patients (86%). Other clinical characteristics such as disease duration, age, sex distribution, age at disease onset, and levodopa equivalent daily dosage (LEDD) did not differ between PD patients with and without LTF and did not differ between LTF patients with normal and pathological SVV (Table 1).

## 4. Discussion

In the present study, pathological deviation of the subjective visual vertical (SVV) was present in 67% of PD patients with lateral trunk flexion (LTF). Analysis of SVV is a sensitive tool to assess vestibular tone in the roll plane [4].

Early studies showed that SVV processing involves numerous levels of the central nervous system; for example, frontal lobe pathology can result in abnormal SVV [9]. Furthermore, disease duration and degree of cognitive impairment also seem to have an influence on visuospatial processing and could thereby influence SVV angles [10].

However, these studies did not relate their findings to postural abnormality in PD patients. Recent data suggest that peripheral vestibular pathology can be found in PD patients and is at least partially involved in lateral trunk flexion of PD patients [3, 11]. Since deviation of the SVV is indicative of vestibular dysbalance, our results are therefore in line with these findings.

Deviation of SVV towards the side of LTF in patients with PD and Pisa Syndrome has recently been described by Scocco et al. [11]. The present study extends these findings by showing that SVV angles outside the normal range were observed in all subjects with mobile LTF but only in a third of cases with fixed LTF. Furthermore, SVV angles were significantly larger in PD patients with mobile versus fixed LTF. These findings might indicate differences in the pathophysiology of both types of LTF in PD, where vestibular dysbalance might be a key factor in the pathogenesis of mobile lateral flexion, while fixed LTF might primarily involve vertebrogenic or muscular mechanisms. Interestingly, a recent neuropathological study reported alpha-synucleinopathy in the vestibular nuclei complex of patients with PD [12]. Such neurodegenerative changes could account for the peripheral vestibular dysbalance observed in this study and previous studies analyzing vestibular function in PD patients [3, 11]. There are however presently no studies linking regional brainstem synuclein deposits to premortem clinical features.

Primary neurodegeneration in central vestibular structures might cause altered verticality perception in PD patients and alter the “normal value” for vertical. Lateral flexion, congruent with the deviation of the SVV, could reflect the attempt to align the body to the supposed vertical. A contradictory theory would be that deviation of SVV is an epiphenomenon and is secondary to the postural deviation of the trunk, which is caused by other, for example, muscular or vertebrogenic mechanisms [11]. Rather supporting the first hypothesis, Vitale and colleagues described two PD patients without LTF but marked vestibular dysbalance to develop mobile LTF ipsilateral to the vestibular hypofunction during six months of follow-up [3].

Our observations have to be interpreted with caution given the small number of controls in comparison to PD patients with LTF and the confounder of recall bias when retrospectively assessing time of LTF onset. Also, in contrast to Zwergal et al. who introduced the bucket test method and validated the results with ten repeats, in this study means of SVV deviation were calculated from three repeats only [6]. Furthermore, patients with a history of cerebrovascular events or acute vestibular pathology were excluded from this trial, since pathological SVV deviations are also found in these conditions [13, 14]. However, we did not perform routine cerebral imaging to screen for structural cerebral damage. Thus, we cannot fully exclude structural causes for SVV deviation, despite missing clinical evidence for focal neurological deficits.

Despite the study limitations, we propose an interesting hypothesis for future studies in a larger cohort and recommend testing PD patients with LTF for vestibular dysbalance utilizing SVV analysis with a brief, inexpensive, and noninvasive bedside test. Therapeutic approaches targeting the vestibular system such as modified Cawthorne and Cooksey training or neurofeedback methods with vibrotactile feedback signals [15] could be considered in PD patients with vestibular dysbalance. Future studies should correlate impaired visuospatial perception and cognition [10] to pathological SVV angles.

## Figures and Tables

**Table 1:** Comparison of clinical features and demographic factors in PD patients with and without LTF (controls) and PD patients with LTF.

	Controls	LTF			*p* values	
		All	Normal SVV	Pathological SVV	Controls versus all LTF	Normal SVV versus pathological SVV
*n*	9	21	7	14		
SVV angles (degree)	0.8 (0.1–1.9)	4.3 (0.1–17.7)	1.2 (0.1–2.0)	6.5 (2.8–17.7)	<0.001^#^	<0.001^#^
LTF type (mobile/fixed)	n/a	10/11	0/7	10/4	n.a.	<0.01^+^
LTF onset (before PD/after PD)	n/a	10/11	0/7	10/4	n.a.	<0.01^+^
LTF angles (degree)	n/a	16 (10–48)	10 (10–21)	14 (10–48)	n.a.	0.56^#^
LTF (R/L)	n/a	14/7	3/4	11/3	n.a.	0.16^+^
PD onset (R/L/bil)	5/3/1	10/11/0	2/5/0	8/6/0	0.23^&^	0.36^+^
Age (years)	71 ± 8	69 ± 7	70 ± 8	69 ± 6	0.53^§^	0.79^§^
Sex (m/f)	8/1	11/10	3/4	8/6	0.10^+^	0.66^+^
Age at onset (years)	62 ± 12	58 ± 7	61 ± 7	56 ± 7	0.37^§^	0.15^§^
Disease duration (years)	10 ± 9	12 ± 6	10 ± 4	13 ± 7	0.55^§^	0.32^§^
LEDD (mg)	725 (300–1260)	1023 (330–2333)	1070 (725–2333)	850 (330–2070)	0.15^#^	0.10^#^

Mean ± SD or median (range). ^#^Mann-Whitney *U* test, ^+^Fisher's exact test, ^&^Chi square test, and ^§^Welch's *t*-test. Median (range) or mean ± SD. R: right; L: left; bil: bilateral; LEDD: levodopa equivalent daily dosage.

## References

[1] Doherty K. M., van de Warrenburg B. P., Peralta M. C. (2011). Postural deformities in Parkinson's disease. *The Lancet Neurology*.

[2] Doherty K. M., Davagnanam I., Molloy S., Silveira-Moriyama L., Lees A. J. (2013). Pisa syndrome in Parkinson's disease: a mobile or fixed deformity?. *Journal of Neurology, Neurosurgery and Psychiatry*.

[3] Vitale C., Marcelli V., Furia T. (2011). Vestibular impairment and adaptive postural imbalance in parkinsonian patients with lateral trunk flexion. *Movement Disorders*.

[4] Dieterich M., Brandt T. (1993). Ocular torsion and tilt of subjective visual vertical are sensitive brainstem signs. *Annals of Neurology*.

[5] Jeon J. Angle Meter Version 3.0. https://itunes.apple.com.

[6] Zwergal A., Rettinger N., Frenzel C., Dieterich M., Brandt T., Strupp M. (2009). A bucket of static vestibular function. *Neurology*.

[7] http://itunes.apple.com.

[8] Schönfeld U., Clarke A. H. (2011). A clinical study of the subjective visual vertical during unilateral centrifugation and static tilt. *Acta Oto-Laryngologica*.

[9] Proctor F., Riklan M., Cooper I. S., Teuber H. L. (1964). Judgement of visual and postural vertical by parkinsonian patients. *Neurology*.

[10] Raskin S. A., Borod J. C., Wasserstein J., Bodis-Wollner I., Coscia L., Yahr M. D. (1990). Visuospatial orientation in parkinson's disease. *International Journal of Neuroscience*.

[11] Scocco D. H., Wagner J. N., Racosta J., Chade A., Gershanik O. S. (2014). Subjective visual vertical in Pisa syndrome. *Parkinsonism and Related Disorders*.

[12] Seidel K., Mahlke J., Siswanto S. (2014). The brainstem pathologies of Parkinson's disease and dementia with lewy bodies. *Brain Pathology*.

[13] Kim H.-A., Hong J.-H., Lee H. (2008). Otolith dysfunction in vestibular neuritis: recovery pattern and a predictor of symptom recovery. *Neurology*.

[14] Yang T.-H., Oh S.-Y., Kwak K., Lee J.-M., Shin B.-S., Jeong S.-K. (2014). Topology of brainstem lesions associated with subjective visual vertical tilt. *Neurology*.

[15] Basta D., Rossi-Izquierdo M., Soto-Varela A. (2011). Efficacy of a vibrotactile neurofeedback training in stance and gait conditions for the treatment of balance deficits: a double-blind, placebo-controlled multicenter study. *Otology and Neurotology*.

